# Granuloma-in-follicles in pediatric Crohn’s disease

**DOI:** 10.1186/1546-0096-9-S1-P294

**Published:** 2011-09-14

**Authors:** Carl EI Janssen, Isabelle Cleynen, Gert De Hertogh, Valeer J Desmet, Tania Roskams, Carine H Wouters

**Affiliations:** 1 Departments of Pathology, University Hospital Leuven, Belgium; 2 Gastro-enterology, University Hospital Leuven, Belgium; 3 Pediatric Rheumatology, University Hospital Leuven, Belgium

## Background

SNPs in NOD2 are a major susceptibility factor for Crohn’s disease (CD). (Sub)mucosal granulomas are a diagnostic feature of CD.

## Aim

To better describe morphological and immunohistochemical featurs of pediatric CD (pCD) granulomas.

## Methods

**Figure 1:**
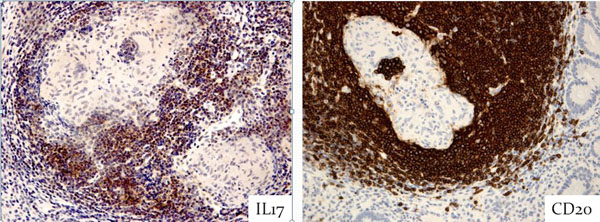


17 pCD patients with granulomas were genotyped for CD-associated mutations or polymorphisms in NOD2 and autophagy-related genes ATG1 and ATG16L1. Granulomas were found in intestinal biopsies of 22 out of 83 endoscopic and 9 out of 12 surgical procedures. Of each procedure we selected one biopsy per organ to study the cellular composition and cytokine profile of granulomas. We stained 43 paraffin-embedded, formalin-fixed pCD biopsies with hematoxylin&eosin and monoclonal antibodies targeting leukocyte markers (HLA-DR, CD68, CD4, CD8, CD20, IL23R) and cytokines (TNFα, IFNγ, IL6, IL10, IL17, TGFβ) (Figure 1) Morphology and immunohistochemistry were scored semi-quantitatively.

## Results

In addition to small isolated (sub)mucosal granulomas, we found granulomas in the follicle(GIF) centre of mucosa-associated lymphoid tissue, visualized by CD20 staining (fig). GIF were found in half the number of procedures (more often in chirurgical than in endoscopic) in only 7 out of 17 pCD patients. Exceptional lymphocyte emperipolesis in multinucleated giant cells (MGCs) and IL-17 expression were observed, only in granulomas of pCD with GIF (fig). The granulomas of patients with GIF often had a lymphocytic corona, but polycyclic granuloma architecture was rare. No clear relation was seen between the presence of GIF and the CD-associated NOD2, ATG1 and ATG16L1 genotypes.

## Conclusion

We defined GIF as a morphological characteristic in few selected pCD patients, and demonstrate association with emperipolesis of lymphocytes in MGCs and weak IL17 expression.

